# The interference of COVID-19 in the male reproductive system: Important questions and the future of assisted reproduction techniques

**DOI:** 10.6061/clinics/2020/e2183

**Published:** 2020-08-21

**Authors:** Renata Cristina de Carvalho, Matheus Ferreira Groner, Jacqueline Camillo, Paulo Roberto Abrão Ferreira, Renato Fraietta

**Affiliations:** I Disciplina de Urologia, Departamento de Cirurgia, Universidade Federal de Sao Paulo (UNIFESP), Sao Paulo, SP, BR.; II Disciplina de Infectologia, Departamento de Medicina, Universidade Federal de Sao Paulo (UNIFESP), Sao Paulo, SP, BR.

## COVID-19 AND SEMEN

Due to the pandemic caused by the novel coronavirus disease, researchers internationally have started to utilize their efforts in understanding its pathophysiology and method of action (1). Ongoing studies have reported that severe acute respiratory syndrome coronavirus 2 (SARS-Cov-2) is easily found in most human bodily fluids (2-4). In relation to semen, there is great interest in unraveling the possible interaction between this fluid sample and the SARS-Cov-2 microorganism, as well as the possible long term consequences of this relationship (5). Perhaps the most significant dimension of this theme is related to our current knowledge of other viral infections, in which the causative agents are found in the seminal sample, which use this medium for their transmission while causing alterations in the fertile potential of their carriers (6-9).

In addition, the form of the SARS-Cov-2 infection that has been established in the literature, and its mechanism of action, are similar to that of the SARS-Cov virus (10). Using the cellular receptor angiotensin converting enzyme 2 (ACE2), this virus infiltrates the cells where it then starts its multiplication (11,12). Interestingly, these receptors are found in high concentrations within the germ and somatic cells of the human testicles (13); however, recent studies show that, for the cell infection process to be successful, the transmembrane serine protease 2 (TMPRSS2) protein that assists in the virus-cell fusion process needs to be present (14). However, the expression of this molecule is rarely found within testicular tissue (15). Thus, there are doubts as to whether the testis is an organ that is vulnerable to this new infection.

## QUESTIONS TO BE ANSWERED

Taking into account all this information, several issues around the Coronavirus to semen and male fertility relationships are being raised, including:

- Can SARS-Cov-2 be found in semen?
- Is the seminal sample a means of transmission for this virus?
- Can this viral infection lead to loss of gonadal function or changes in male fertility potential?
- If this loss or change is proven, is it a reversible or permanent?
- In relation to assisted reproduction techniques (ART), will future conduct in this area need to be reprogrammed?

Some studies have attempted to answer these questions; however, they contain biases that can alter their results, such as a low number of sample patients or due to the moment when the analyses were performed (e.g., acute phase of the infection, in recovery, or already recovered) (16-18).

## FUTURE FOLLOWING THE COVID-19 PANDEMIC

The importance of obtaining these answers lies in the fact that there is a need for a change of conduct in the face of the new reality imposed by the novel coronavirus. If the presence of SARS-Cov-2 in semen is confirmed, the methods of assisted human reproduction conduct should be modified, ensuring the timely safety of couples; however, current information about the virus raises other issues, such as: if seminal transmission exists, should a couple avoid sexual intercourse or use a barrier method if the male partner is known to be positive for the coronavirus disease 2019 (COVID-19)? As for ART, should we test all men for COVID-19 just as we do for HIV, hepatitis B and C, syphilis, HTLV I/II, and Zika before the procedure? If there is SARS-Cov-2 in an infected male’s semen, is double sperm washing effective in isolating the virus as it is for HIV and hepatitis C? Should we take special care when cryopreserving semen from COVID-19 positive men? These questions’ answers will have a direct impact on the daily routine of Andrologists and Reproductive Endocrinologists.

In addition, even with the absence of the virus in the seminal sample, a study has reported the presence of orchialgia in men diagnosed with COVID-19 (16), which is indicative of testicular damage. Moreover, a frequent symptom of this infection is fever (19), which alone is known as causing spermatogenesis damage due to this process’ sensitivity to heat (20,21). This increases our concerns about male fertility. Thus, it is important to include the presence of a previous diagnosis of COVID-19 in the patient’s anamnesis to deepen our fertility investigation because, if there is a proven interference of the virus in the male fertility potential, alternative treatments should be studied.

## CONCLUSIONS

In conclusion, the COVID-19 pandemic has brought many uncertainties to the future of ART and male infertility management. Additional studies are necessary to answer all these questions; however, while we do not currently have all the answers, the most effective conduct is to be cautious and evaluate each reproduction treatment step individually when dealing with a couple in which the man has been diagnosed with COVID-19.

## AUTHOR CONTRIBUTIONS

De Carvalho RC wrote the manuscript and conceived this research. Groner MF, Camillo J, Ferreira PRA analyzed the topic in the literature. Fraietta R conceived the study and coordinated this research. All authors revised and accepted the final version of the manuscript.
